# Childhood trauma and disordered eating behaviors in youth: examining individual types, cumulative numbers, and latent patterns

**DOI:** 10.1186/s13034-025-00928-y

**Published:** 2025-08-01

**Authors:** Yan’e Lu, Wenwen Xu, Suying Wu, Liangliang Ping, Qingyan Wu, Yuyun Huang, Li Zhang, Farong Liu, Jia Jia Liu, Jianyu Que

**Affiliations:** 1 School of Nursing, Peking University, 38 Xueyuan Road, Haidian District, Beijing, 100191 China; 2 Xiamen Xianyue Hospital, Xianyue Hospital Affiliated with Xiamen Medical College, Fujian Psychiatric Center, Fujian Clinical Research Center for Mental Disorders, 387-399 Xianyue Road, Siming District, Xiamen, 361012 Fujian China; 3 Fujian Medical University, Fuzhou, Fujian China; 4 Mental Health Institute of Inner Mongolia Autonomous Region, Hohhot, Inner Mongolia China

**Keywords:** Childhood trauma, Disordered eating, Youth, Cumulative score, Latent class analysis

## Abstract

**Background:**

Although traumatic experiences in childhood have been recognized as contributors to disordered eating behaviors, critical gaps remain in our understanding. There is limited evidence on the individual, cumulative, and distinct patterns of childhood trauma associated with risky restrictive eating and binge/purging behaviors.

**Methods:**

Individuals aged 12–25 years from Xiamen, Fujian Province, China, completed online questionnaires assessing childhood trauma, risky restrictive eating and binge/purging behaviors. Childhood trauma was examined through three analytical approaches: individual types, cumulative trauma scores, and latent class analysis. Logistic regression models were used to investigate the associations between childhood trauma and disordered eating behaviors.

**Results:**

Among the 3424 participants, 7.3% (*n* = 251) reported disordered eating behaviors occurring two or more times per month over the past year. After adjusting for covariates, our analyses showed that emotional abuse independently associated with increased odds of both risky restrictive eating (OR: 2.11, 95% CI 1.28–3.47) and binge/purging behaviors (OR: 2.20, 95% CI 1.34–3.62). Physical abuse was associated only with increased odds of binge/purging behavior (OR: 2.09, 95% CI 1.20–3.64). Traumatic experiences showed a cumulative effect on disordered eating, significant at two or more incidents. Three trauma patterns were identified: ‘low trauma,’ ‘low neglect,’ and ‘high trauma.’ Youth with the ‘high trauma’ pattern exhibited the highest odds of both risky restrictive eating (OR: 2.88, 95% CI 1.65–5.02) and binge/purging behaviors (OR: 3.21, 95% CI 1.85–5.54), whereas those in the ‘high neglect’ pattern showed increased odds only for binge/purging behavior (OR: 1.52, 95% CI 1.01–2.29).

**Conclusions:**

Our findings highlight the need to consider the types, numbers, and patterns of childhood trauma when developing prevention and treatment strategies for disordered eating behaviors.

**Supplementary Information:**

The online version contains supplementary material available at 10.1186/s13034-025-00928-y.

## Introduction

Disordered eating behaviors—such as risky restrictive eating and binge/purging—are maladaptive coping strategies that significantly impair psychological, social, and physical functioning [1–3]. These behaviors often emerge during adolescence, preceding the onset of clinically diagnosable eating disorders, and serve as crucial early indicators of later psychopathology [4, 5]. Longitudinal evidence has shown that adolescents who engage in disordered eating behaviors are at increased risk for subsequent smoking, substance use, and self-harm in young adulthood [6]. Furthermore, an umbrella review has linked these behaviors to an increased risk of anxiety, depression, and suicidal ideation [7]. Thus, identifying risk factors for disordered eating behaviors in youth is essential for informing early prevention efforts.

Childhood trauma—typically including emotional abuse, physical abuse, emotional neglect, physical neglect, and sexual abuse—has been recognized as a significant contributor to disordered eating behaviors [4]. Theoretical frameworks such as the Developmental Trauma Model [8] and the Dual Pathway Model [9, 10] offer valuable insights into the underlying mechanisms of this association. Specifically, the Developmental Trauma Model posits that early interpersonal trauma disrupts affect regulation, bodily awareness, and identity formation [8], which are domains frequently impaired in individuals with disordered eating [11]. Meanwhile, the Dual Pathway Model suggests that negative affect and body dissatisfaction, both of which may be consequences of early trauma [12], drive maladaptive eating patterns, such as binge eating, purging, and restrictive dieting [9, 10].

Empirical research has begun to explore the specific links between individual trauma types and disordered eating behaviors [13, 14]. Recent evidence suggests that different types of childhood trauma may be differentially associated with specific disordered eating behaviors [15, 16]. Two meta-analyses provide key support for this proposition. Caslini et al. found that while all forms of childhood abuse increased the risk of eating disorders, only bulimia nervosa and binge eating disorder were consistently associated with emotional, physical, and sexual abuse, whereas anorexia nervosa was linked only to physical abuse [15]. Molendijk et al. further reported stronger associations of childhood trauma with bulimia nervosa, binge eating disorder, and the binge/purge subtype of anorexia nervosa, but weaker associations with the restricting subtype [16]. These findings underscore the need to consider both trauma type and disordered eating subtype when examining trauma–eating pathology associations. However, the independent effects of specific trauma types have been reported inconsistently [13, 14], partly due to methodological limitations. Most existing studies adjust only for demographic variables, overlooking well-established psychological contributors to disordered eating, such as depression [17], anxiety [18], and insomnia [19]. This omission may bias effect estimates and obscure the specificity of associations between trauma and disordered eating behaviors. Therefore, studies that simultaneously consider multiple trauma types and comprehensively adjust for relevant psychological covariates are needed to clarify these differential effects.

Another important but underexplored area concerns the cumulative and patterned nature of childhood trauma. In real-world settings, trauma rarely occurs in isolation [20]. Youth frequently experience overlapping forms of abuse or neglect, and cumulative trauma exposure has been linked to greater psychiatric vulnerability [21, 22]. While several studies have examined cumulative trauma effects on disordered eating behaviors, the existing evidence primarily stems from clinical [16] or Western samples [13], limiting generalizability to broader and more diverse populations. Furthermore, trauma exposures tend to cluster in non-random, heterogeneous patterns [23, 24]. Latent class analysis (LCA) has emerged as a valuable tool for identifying these patterns. Previous findings suggest that trauma patterns characterized by multiple forms of abuse may be particularly harmful for specific health outcomes [25, 26], including disordered eating behaviors [27]. Nonetheless, little is known about how these latent trauma patterns are specifically associated with distinct disordered eating subtypes, such as risky restrictive eating and binge/purging behaviors.

In light of these gaps, the present study aims to make several novel contributions to the existing literature. First, we adopt a comprehensive analytic strategy that simultaneously examines three operationalizations of childhood trauma—individual trauma types, cumulative trauma exposure, and latent trauma patterns—in relation to two specific disordered eating behaviors: risky restrictive eating and binge/purging. Second, we adjust for a broad range of relevant covariates, including both demographic characteristics and key psychological symptoms (e.g. depression, anxiety, insomnia), thereby helping to mitigate concerns about residual confounding in prior work. Third, this study focuses on a relatively large, non-clinical sample of Chinese youths, a population underrepresented in trauma-eating behavior research. In summary, these efforts aim to provide a more nuanced understanding of how different trauma experiences contribute to distinct disordered eating behaviors, thereby offering empirical insights to inform trauma-informed prevention and intervention strategies for youth.

## Methods

### Participants

This cross-sectional study was conducted using the online platform “Questionnaire Star” from December 15, 2022, to May 26, 2023. Participants were youths aged 12–25 years residing in Xiamen, Fujian Province, China. Recruitment was primarily conducted through digital posters disseminated by schoolteachers via school-based WeChat chat groups of students and their parents. Additionally, some teachers and researchers shared the recruitment materials on their personal social media accounts (e.g., WeChat Moments), potentially reaching individuals outside the formal school system. Interested participants could access the survey by scanning a QR code on the poster. This convenient recruitment strategy likely favored the inclusion of school-enrolled youths with access to digital school communication platforms.

To ensure data quality, a basic math question (“What is the result of 21 minus 7?”) and an authentication item were included. Prior to participation, all respondents provided electronic informed consent. For those under 18, consent from legal guardians was required and obtained through parental WeChat groups. Of the 7279 initial respondents, 6145 were retained after excluding 481 for incorrect math answers and 653 for inconsistent or insincere responses. The final analytic sample comprised 3424 youths who completed all items assessing childhood trauma, risky restrictive eating, and binge/purging behaviors. The study protocol was approved by the Ethics Committee of Xiamen Xianyue Hospital (2022-KY-26).

### Measures

#### Childhood trauma

Childhood trauma in youths was assessed using the Chinese version of Childhood Trauma Questionnaire-Short Form (CTQ-SF) [28], which demonstrated high internal consistency (Cronbach’s alpha = 0.85) in the present study. The CTQ-SF includes five types of trauma: emotional abuse, physical abuse, sexual abuse, emotional neglect, and physical neglect [28]. Participants rated each item on a 5-point Likert scale, ranging from “1 = never true” to “5 = very often true”. Consistent with previous research, the thresholds for a history of abuse or neglect were set as follows: emotional abuse ≥ 13, physical abuse ≥ 10, sexual abuse ≥ 8, emotional neglect ≥ 15, and physical neglect ≥ 10 [29, 30].

#### Disordered eating behaviors

Although standardized instruments such as the Eating Disorder Examination Questionnaire and Eating Attitudes Test are commonly used to assess the severity of disordered eating, their length and complexity may limit feasibility in large-scale epidemiological studies. In contrast, brief items derived from validated inventories offer a practical alternative, enabling efficient screening while maintaining acceptable face validity. This approach has been widely adopted in large-scale youth surveys to identify disordered eating behaviors [31, 32]. Accordingly, in the present study, disordered eating behaviors were assessed using two specific items related to unhealthy dietary behaviors from the Adolescent Health-related Risky Behavior Inventory [33, 34], a validated tool that assesses the frequency of 38 health-risk behaviors over the past year. Specifically, participants responded on a 5-point scale: “never” scored 0, “hardly ever” (once per month) scored 1, “sometimes” (two to four times per month) scored 2, “often” (two to three times per week) scored 3, and “always” (more than four times per week) scored 4. Risky restrictive eating behavior was measured by: “Have you ever engaged in excessive dieting that caused physical discomfort such as dizziness, cold sweats, or lack of energy?” Binge/purging behavior was measured by: “Have you ever engaged in binge eating or vomited after binge eating?”, which captures both behaviors without distinguishing between them as separate clinical constructs [34]. Both risky restrictive eating and binge/purging behaviors were operationally defined as occurring at least twice per month, corresponding to a score of 2 or higher.

#### Covariates

Covariates were selected based on theoretical relevance and prior empirical evidence [4, 35, 36], including demographic characteristics and psychological symptoms. Demographic characteristics included age, sex, body mass index (BMI), exercise frequency, smoking behaviors, drinking behaviors, paternal and maternal education levels, as well as parental psychological and sleep problems. Psychological symptoms such as depression, anxiety, and insomnia were assessed using the 9-item Patient Health Questionnaire (PHQ-9) [37], the 7-item Generalized Anxiety Disorder Scale [38], and the Insomnia Severity Index [39], respectively. To reduce construct overlap and collinearity, items related to sleep disturbances and appetite changes were omitted from the PHQ-9, resulting in a 7-item depression score [40]. Notably, given our primary aim to examine the direct association between childhood trauma and disordered eating behaviors, the adjustment set encompassed both potential confounders (e.g., age, sex, BMI, parental education, parental psychological and sleep problems) and potential mediators (e.g., depression, anxiety, insomnia symptoms). This strategy reflects a conservative approach to minimize potential residual confounding and enhance the robustness of the findings, despite the possibility of attenuating the estimated total effect. The hypothesized relationships among childhood trauma, disordered eating behaviors, and covariates were further depicted using a Directed Acyclic Graph (Figure S1).

### Statistical analysis

Statistical analysis was performed using SPSS 26.0 and Mplus 8.3, with a two-sided *p* < 0.05 considered statistically significant. Continuous variables were reported as means (M) ± standard deviation (SD) for normally distributed data, and as median with interquartile range (IQR) for non-normally distributed data. Categorical variables were presented as frequencies (n) and percentages (%).

Firstly, childhood trauma was operated using three approaches: individual trauma types, cumulative scores, and latent patterns. To evaluate the independent effects of each trauma type, logistic regression models adjusted for other trauma types were conducted to isolate the unique contribution of each type. The cumulative trauma score was calculated by summing the number of trauma types experienced. LCA was applied to identify the latent patterns of childhood traumas, creating models with 1–5 classes. The best class solution was determined by model fit statistics and clinical interpretability. Specifically, lower values of the Akaike Information Criterion (AIC), Bayesian Information Criterion (BIC), and sample-size adjusted BIC indicated better model fit [41]. Entropy values above 0.80 were considered satisfactory for measuring classification accuracy [42]. The Vuong-Lo-Mendell-Rubin likelihood ratio test (LMRT) and the bootstrapped likelihood ratio test (BLRT) were used to assess whether the current model was preferable to a model with one fewer class [43]. Additionally, ANOVA, Kruskal–Wallis, and Chi-square tests were conducted to compare differences in covariates and childhood trauma across LCA-identified trauma patterns.

Secondly, binary logistic regressions were conducted to examine associations between different trauma types, cumulative trauma scores, and LCA-identified trauma patterns with risky restrictive eating and binge/purging behaviors, respectively. For each model, adjusted analyses were conducted without covariates, with demographic characteristics only, and with both demographic characteristics and psychological symptoms. Notably, binge/purging behavior was controlled as a covariate in analyses with risky restrictive eating behavior as the outcome, and vice versa, to account for their frequent co-occurrence and potential bidirectional influence [44].

Finally, to more precisely capture the heterogeneity of disordered eating behaviors, a four-category variable termed ‘disordered eating behaviors’ was created based on whether participants exhibited both risky restrictive eating and binge/purging behaviors. Participants were categorized into four mutually exclusive subgroups: (1) neither behavior, (2) risky restrictive eating only, (3) binge/purging only, and (4) both behaviors. A similar fine-grained classification strategy, which accounts for the potential co-occurrence of distinct disordered eating behaviors, rather than collapsing them into a single composite outcome, has been employed in previous large-scale studies of adolescents and young adults [31, 32]. To assess the robustness of our findings, multinomial logistic regressions were conducted in supplementary analyses using ‘neither behavior’ and ‘both behaviors’ as the reference categories, respectively.

## Results

### Sample characteristics

Table 1 details the characteristics of the 3424 participants included in the study. The mean age was 18.11 ± 2.26 years, with females constituting 58.4% (*n* = 2000) of the sample. Additionally, 7.3% (*n* = 251) of the participants experienced disordered eating behaviors two or more times per month over the past year. Specifically, 2.8% (*n* = 97) engaged only in risky restrictive eating, 2.9% (*n* = 99) only in binge/purging, and 1.6% (*n* = 55) reported both risky restrictive eating and binge/purging behaviors.

**Table 1:** Sample characteristics (*N*=3424)

Variables	M (SD)/*n* (%)/median (IQR)
Age	18.11 (2.26)
Sex	
Male	1424 (41.6)
Female	2000 (58.4)
BMI	
Underweight	869 (25.4)
Normal	1968 (57.5)
Overweight	587 (17.1)
Exercise frequency	
No	1423 (41.6)
1–3 days/week	1327 (38.8)
>3 days/week	674 (19.7)
Smoking behavior	
No	3185 (93.0)
Yes	218(6.4)
Missing value	21 (0.6)
Drinking behavior	
No	2752 (80.4)
Yes	669 (19.5)
Missing value	3 (0.1)
Paternal education level	
Middle school and below	1643 (48.0)
Senior high school	894 (26.1)
College degree and above	887 (25.9)
Maternal education level	
Middle school and below	1933 (56.5)
Senior high school	825 (24.1)
College degree and above	666 (19.5)
Parental psychological problems	
No	3077 (89.9)
Yes	347 (10.1)
Parental sleep problems	
No	2484 (72.5)
Yes	940 (27.5)
Depression symptoms	6.00 (8.00)
Anxiety symptoms	4.00 (7.00)
Insomnia symptoms	6.00 (8.00)
Childhood trauma total score	40.17 (10.95)
Risky restrictive eating behavior	
No	3272 (95.6)
Yes	152 (4.4)
Binge/purging behavior	
No	3270 (95.5)
Yes	154 (4.5)
Disordered eating behavior	
Neither behavior	3173 (92.7)
Risky restrictive eating only	97 (2.8)
Binge/purging only	99 (2.9)
Both behaviors	55 (1.6)

*M* mean, *SD* standard deviation. *n* Frequencies; % Percentages, *IQR* interquartile range, *BMI* Body mass index

### Individual, cumulative, and LCA-identified childhood trauma types

As shown in Table 2, 7.1% of participants reported emotional abuse, exceeding rates of physical abuse (4.6%) and sexual abuse (4.4%). The predominant forms of traumas were physical neglect (38.8%, *n* = 1330) and emotional neglect (35.8%, *n* = 1225). Additionally, 23.1% (*n* = 792) of the participants experienced one type of trauma, while 28.9% (*n* = 988) faced two or more types.

**Table 2:** Individual, cumulative, and LCA-identified childhood trauma types

Childhood trauma types	*n* (%)
Individual childhood trauma types	
Emotional abuse	
No	3181 (92.9)
Yes	243 (7.1)
Physical abuse	
No	3265 (95.4)
Yes	159 (4.6)
Sexual abuse	
No	3272 (95.6)
Yes	152 (4.4)
Emotional neglect	
No	2199 (64.2)
Yes	1225 (35.8)
Physical neglect	
No	2094 (61.2)
Yes	1330 (38.8)
Cumulative childhood trauma types	
0	1644 (48.0)
1	792 (23.1)
2	767 (22.4)
≥3	221 (6.5)
LCA-identified childhood trauma types	
Low trauma	2179 (63.6)
High neglect	1096 (32.0)
High trauma	149 (4.4)

*n*=Frequencies; %= percentages; LCA=latent class analysis

**Fig. 1:**
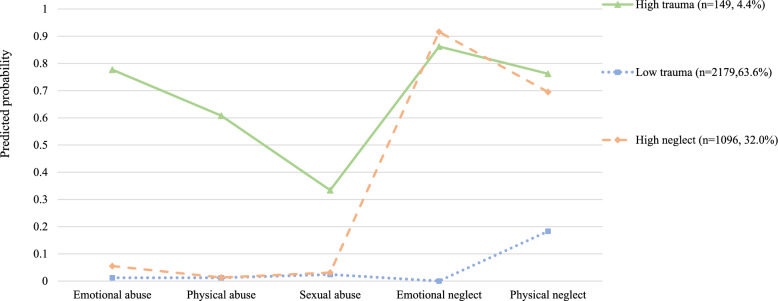
Proportion of youth with five different types of childhood trauma for the three-class solution (*N* = 3424)

Three patterns of childhood trauma were identified based on model fitting statistics (Tables S1 and S2). The majority of participants (63.6%, *n* = 2179) were less likely to report any traumas, categorized as ‘low trauma.’ 32.0% (*n* = 1096) exhibited a lower likelihood of abuse but higher rates of neglect, labeled as ‘high neglect.’ A minority (4.4%, *n* = 149) faced a higher probability of experiencing all types of traumas, referred to as ‘high trauma’ (Fig. 1). Specifically, individuals in the ‘high trauma’ patterns showed significantly higher rates of having parents with psychological and sleep issues, as well as increased personal symptoms of depression, anxiety, and insomnia compared to those in ‘low trauma’ and ‘high neglect’ (Tables S3).

### Associations between childhood trauma and risky restrictive eating behavior

As shown in Table 3, emotional abuse, sexual abuse, and physical neglect were significantly associated with increased odds of risky restrictive eating behavior in the unadjusted model. However, after further adjustment for demographic characteristics and psychological symptoms, only emotional abuse retained a significant association (OR: 2.11, 95% CI 1.28–3.47). A dose–response trend was observed with the cumulative number of trauma types: compared with individuals without trauma exposure, those with one, two, or three or more types of trauma were associated with progressively higher odds of risky restrictive eating behavior in both the unadjusted and demographic-adjusted models. After adjustment for psychological symptoms, exposure to two (OR: 1.83, 95% CI 1.08–3.11) or three or more trauma types (OR: 2.77, 95% CI 1.56–4.93) remained significantly associated. The odds of engaging in risky restrictive eating behaviors were significantly higher among individuals with a ‘high trauma’ pattern compared to those with ‘low trauma’ and ‘high neglect’ patterns across both unadjusted and adjusted models. Nevertheless, after full adjustment, no statistically significant difference was observed between the ‘low trauma’ and ‘high neglect’ patterns (OR: 1.46, 95% CI 0.96–2.23). The results of the supplementary multiple logistic regression analyses supported these findings (Tables S4 and S5).

**Table 3:** Association of childhood trauma with risky restrictive eating behaviour

Variables	Model 1		Model 2		Model 3	
	OR (95%CI)	*p*	OR (95%CI)	*p*	OR (95%CI)	*p*
Individual childhood trauma types						
Emotional abuse						
No	Reference		Reference		Reference	
Yes	5.40 (3.42, 8.55)	<0.001	3.81 (2.36, 6.15)	<0.001	2.11 (1.28, 3.47)	0.004
Physical abuse						
No	Reference		Reference		Reference	
Yes	1.60 (0.93, 2.74)	0.090	1.21 (0.68, 2.13)	0.515	1.25 (0.69, 2.26)	0.456
Sexual abuse						
No	Reference		Reference		Reference	
Yes	2.04 (1.20, 3.46)	0.009	1.69 (0.96, 2.97)	0.069	1.49 (0.82, 2.70)	0.194
Emotional neglect						
No	Reference		Reference		Reference	
Yes	1.32 (0.88, 2.00)	0.181	1.29 (0.84, 1.98)	0.243	1.14 (0.73, 1.78)	0.566
Physical neglect						
No	Reference		Reference		Reference	
Yes	1.70 (1.14, 2.54)	0.009	1.51 (0.99, 2.30)	0.053	1.19 (0.77, 1.83)	0.430
Cumulative childhood trauma types						
0	Reference		Reference		Reference	
1	2.05 (1.22, 3.43)	0.007	1.76 (1.04, 2.99)	0.036	1.33 (0.76, 2.31)	0.314
2	3.20 (1.99, 5.14)	<0.001	2.93 (1.79, 4.80)	<0.001	1.83 (1.08, 3.11)	0.025
≥3	15.73 (9.74, 25.41)	<0.001	7.27 (4.29, 12.33)	<0.001	2.77 (1.56, 4.93)	0.001
LCA-identified childhood trauma types						
Low trauma	Reference		Reference		Reference	
High neglect	2.36 (1.62, 3.44)	<0.001	2.10 (1.42, 3.11)	<0.001	1.46 (0.96, 2.23)	0.080
High trauma	13.73 (8.68, 21.72)	<0.001	6.25 (3.74, 10.43)	<0.001	2.88 (1.65, 5.02)	<0.001
High neglect	Reference		Reference		Reference	
High trauma	5.81 (3.70, 9.11)	<0.001	2.98 (1.81, 4.91)	<0.001	1.98 (1.15, 3.38)	0.013

*LCA* latent class analysis; Reference=1; CI=confidence interval; Model 1: unadjusted covariates; Model 2: adjusted for age, sex, BMI, exercise frequency, smoking behavior, drinking behavior, paternal and maternal education level, parental psychological and sleep problems, and binge/purging behavior; Model 3: adjusted for age, sex, BMI, exercise frequency, smoking behavior, drinking behavior, paternal and maternal education level, parental psychological and sleep problems, depression symptoms, anxiety symptoms, insomnia symptoms, and binge/purging behavior

### Associations between childhood trauma and binge/purging behavior

As presented in Table 4, emotional abuse, physical abuse, and emotional neglect were significantly associated with increased odds of binge/purging behavior in the unadjusted model. After adjustment for demographic characteristics and psychological symptoms, the odds for emotional abuse (OR: 2.20, 95% CI 1.34–3.62) and physical abuse (OR: 2.09, 95% CI 1.20–3.64) were attenuated but remained statistically significant. Similarly, a dose–response relationship was also observed between the number of trauma types experienced and the odds of binge/purging behavior after adjustment for covariates. Individuals classified into the ‘high neglect’ (OR: 1.52, 95% CI 1.01–2.29) and ‘high trauma’ (OR: 3.21, 95% CI 1.85–5.54) patterns showed significantly greater odds of binge/purging behavior compared to those in the ‘low trauma’ pattern after full adjustment for covariates. Furthermore, individuals in the ‘high trauma’ pattern were associated with significantly higher odds of binge/purging behavior compared to those in the ‘high neglect’ pattern (OR: 2.11, 95% CI 1.24–3.59). Supplementary analyses were consistent with these findings (Tables S4 and S5).

**Table 4:** Association of childhood trauma with binge/purging behavior

Variables		Model 1		Model 2		Model 3	
		OR (95%CI)	*p*	OR (95%CI)	*p*	OR (95%CI)	*p*
Individual childhood trauma types							
Emotional abuse							
No		Reference		Reference		Reference	
Yes		4.87 (3.07, 7.72)	<0.001	4.01 (2.47, 6.50)	<0.001	2.20 (1.34, 3.62)	0.002
Physical abuse							
No		Reference		Reference		Reference	
Yes		2.64 (1.58, 4.41)	<0.001	2.08 (1.21, 3.58)	0.008	2.09 (1.20, 3.64)	0.009
Sexual abuse							
No		Reference		Reference		Reference	
Yes		1.35 (0.77, 2.39)	0.296	1.09 (0.59, 2.01)	0.782	1.03 (0.55, 1.96)	0.918
Emotional neglect							
No		Reference		Reference		Reference	
Yes		1.54 (1.02, 2.32)	0.038	1.49 (0.97, 2.27)	0.067	1.29 (0.84, 1.99)	0.248
Physical neglect							
No		Reference		Reference		Reference	
Yes		1.13 (0.76, 1.67)	0.552	1.16 (0.76, 1.76)	0.493	0.93 (0.61, 1.43)	0.750
Cumulative childhood trauma types							
0		Reference		Reference		Reference	
1		1.24 (0.73, 2.12)	0.429	1.13 (0.65, 1.96)	0.671	0.84 (0.48, 1.49)	0.552
2		2.90 (1.87, 4.49)	<0.001	2.94 (1.87, 4.64)	<0.001	1.86 (1.15, 3.02)	0.012
≥3		11.73 (7.42,18.55)	<0.001	7.18 (4.30, 12.00)	<0.001	2.88 (1.65, 5.02)	<0.001
LCA-identified childhood trauma types							
Low trauma		Reference		Reference		Reference	
High neglect		2.32 (1.60, 3.35)	<0.001	2.17 (1.48, 3.19)	<0.001	1.52 (1.01, 2.29)	0.045
High trauma		12.76 (8.07, 20.17)	<0.001	6.86 (4.11, 11.47)	<0.001	3.21 (1.85, 5.54)	<0.001
High neglect		Reference		Reference		Reference	
High trauma		5.51 (3.51, 8.65)	<0.001	3.16 (1.92, 5.22)	<0.001	2.11 (1.24, 3.59)	0.006

*LCA* latent class analysis; Reference=1; CI=confidence interval; Model 1: unadjusted covariates; Model 2: adjusted for age, sex, BMI, exercise frequency, smoking behavior, drinking behavior, paternal and maternal education level, parental psychological and sleep problems, and risky restrictive eating behavior; Model 3: adjusted for age, sex, BMI, exercise frequency, smoking behavior, drinking behavior, paternal and maternal education level, parental psychological and sleep problems, depression symptoms, anxiety symptoms, insomnia symptoms, and risky restrictive eating behavior

## Discussion

This study adds to the growing literature by simultaneously examining individual types, cumulative numbers, and distinct latent patterns of childhood trauma in relation to specific disordered eating behaviors in youth, including risky restrictive eating and binge/purging behaviors. Our findings indicated that after accounting for potential covariates, emotional abuse was independently linked to increased odds of both risky restrictive eating and binge/purging behaviors, whereas physical abuse was only associated with higher odds of binge/purging behavior. Individuals exposed to two or more types of traumas faced significantly greater odds for both risky restrictive eating and binge/purging behaviors compared to those without trauma exposure, showing a dose–response trend. Furthermore, we identified three distinct patterns of trauma: ‘low trauma’, ‘high trauma’, and ‘high neglect’. Specifically, youth with the ‘high trauma’ pattern exhibited the highest odds for both risky restrictive eating and binge/purging behaviors, while those in the ‘high neglect’ pattern showed increased odds solely for binge/purging behavior.

In the present study, we observed a prevalence of disordered eating behaviors over 7%, which is slightly lower than the rates reported in other studies conducted in China, where figures that approach or exceed 9% [45, 46]. A likely explanation for this discrepancy is differences in the characteristics of the underlying target populations (e.g., age distribution, educational stage, and geographic region) resulting from varying recruitment strategies. Additionally, demographic variations, such as differences in BMI and gender composition [47, 48] may have contributed to the observed differences. Prior research has indicated that as the average BMI or the percentage of female in the sample increased, the risk of disordered eating behaviors also rose [47, 48]. Furthermore, variations in measurement tools and cut-off scores may also account for the differences in reported prevalence rates. More importantly, systematic reviews have revealed a higher prevalence of screening-detected disordered eating behaviors among high school (13%) [49] and university students (19.7%) [47] globally. These findings emphasize the urgent need to enhance awareness of disordered eating behaviors among the public, parents, and educators.

In this study, emotional abuse was associated with increased odds of disordered eating behaviors, including risky restrictive eating and binge/purging behaviors, aligning with previous findings [50, 51]. One possible explanation for this relationship is the perspective of psycho-developmental processes. Early experiences of emotional abuse have been shown to disrupt emotional regulation, manifesting as emotional inhibition and poor distress tolerance [52]. Specifically, emotional inhibition refers to the excessive suppression of spontaneous behaviors, emotions, or communication, which could appear in eating behaviors as compulsive activities like risky restrictive eating or excessive exercise [52]. Conversely, individuals with low distress tolerance may be more inclined to engage in negative, extreme, and impulsive behaviors when faced with distressing emotions, such as binge/purging behaviors [53, 54]. Moreover, research on the pathways of these associations suggests that emotional dysregulation and maladaptive beliefs stemming from emotional trauma are crucial therapeutic targets for individuals with disordered eating [50, 52, 55]. Both integrative cognitive affective therapy [56] and dialectical behavior therapy [57], which emphasize adaptive emotion regulation strategies, have shown promising results in treating eating disorders.

In the current study, we found that childhood physical abuse was significantly associated with increased odds of binge/purging behaviors, aligning with findings from prior research [58, 59]. Physical abuse may undermine self-esteem and self-efficacy, contributing to negative self-concepts and internalized shame [60]. According to the schema theory of binge eating, low self-esteem and negative self-schemas represent core psychological mechanisms underlying binge eating [61]. Binge/purging behaviors may thus serve as maladaptive coping strategies for psychological escape or self-soothing in response to deeply entrenched negative beliefs about one’s worth, competence, or identity [61]. Additionally, another possible explanation is that the lingering insecurity and life threats from childhood experiences of physical abuse may compel individuals to overeat food as a way to safeguard their physical health [62, 63]. Furthermore, the neuroimmune network hypothesis suggests that early life adversities, like physical abuse, enhance the communication between the brain and the immune system [64]. This interaction fosters chronic low-grade inflammation that influences brain circuits related to threat and reward—specifically, the cortico-amygdala and cortico-basal ganglia pathways—potentially leading individuals to engage in self-medicating behaviors such as smoking, drug use, and the excessive consumption of palatable foods [64].

Similar to previous findings, our study observed a cumulative association between abusive experiences and disordered eating behaviors, with a threshold of two or more incidents [13, 65]. This association persists even when accounting for individual heterogeneity, adopting a person-centered approach over a variable-centered one. Among the three trauma patterns identified in the present study—‘low trauma,’ ‘low neglect,’ and ‘high trauma’—the individuals with ‘high trauma’ pattern showed the highest odds of engaging in risky restrictive eating and binge/purging behaviors. These results support the cumulative risk theory, which posits that exposure to an increasing number of risk factors raises the likelihood of adverse outcomes [66, 67]. Importantly, our findings highlight the necessity in clinical practice to consider not only specific types of traumas but also a history of multiple traumas.

Our findings showed no significant difference in the odds of risky restrictive eating behavior between individuals with a ‘low trauma’ pattern and those with a ‘high neglect’ pattern. However, for binge/purging behaviors, the odds were significantly higher among individuals with a ‘high neglect’ pattern compared to those with a ‘low trauma’ pattern. These results suggest that the ‘high neglect’ pattern effectively differentiates risky restrictive eating from binge/purging behaviors, a novel observation not previously reported in the literature. Therefore, clinicians should prioritize assessing the risk of binge/purging behaviors when individuals report experiencing both types of neglect. Childhood neglect may manifest as food insecurity, stemming from parental disregard for a child’s hunger cues or inadequate provision of food, leading to a restrictive access to food [68]. Research has shown the risk effects of food insecurity on binge behavior [69].

This study has several limitations that should be acknowledged. Firstly, due to its cross-sectional design, causal inferences cannot be drawn from the observed associations. Future longitudinal studies are needed to validate and expand upon these findings. Secondly, disordered eating behaviors were assessed using two single items derived from a reliable but non-clinical scale, rather than a dedicated, validated instrument. While these simplified items offer high face validity and are useful for identifying adolescents at elevated risk in large-scale epidemiological research [33, 34], they may inadequately capture the complexity of disordered eating behaviors and lack diagnostic precision. In particular, the item assessing binge/purging behavior combines both binge eating and purging, representing a double-barreled construct. Future studies should validate our findings using more comprehensive and standardized assessments to improve diagnostic accuracy and capture the full spectrum of disordered eating behaviors. Thirdly, both disordered eating behaviors and childhood trauma were self-reported by participants, potentially introducing recall bias and self-report bias. Fourthly, although this study involved a relatively large sample size, the participants were geographically confined to Xiamen City. Therefore, caution is advised when generalizing these findings to broader populations. Fifthly, the findings were based on a single sample without independent replication, which may limit the robustness and reproducibility of the observed associations. Future research is needed to replicate and validate these findings across diverse and independent cohorts to strengthen their external validity.

## Conclusion

This study contributes to the growing literature by exploring the associations between independent, cumulative, and latent patterns of childhood trauma and specific disordered eating behaviors among youth, including risky restrictive eating as well as binge/purging behaviors. Our findings indicated that emotional abuse was independently associated with increased odds of both risky restrictive eating and binge/purging behaviors, whereas physical abuse was linked only to binge/purging behaviors. Additionally, we observed a cumulative effect of abusive experiences on disordered eating behaviors, with a threshold effect evident at two or more incidents. Furthermore, youth with the ‘high trauma’ pattern exhibited the highest odds for both risky restrictive eating and binge/purging behaviors, while those in the ‘high neglect’ pattern were more likely to report binge/purging behaviors only. These findings underscore the importance of accounting for the heterogeneity in childhood trauma experiences when assessing risk for disordered eating. However, given the descriptive nature of the study, the results should be interpreted with caution. Further research using longitudinal designs and clinically validated assessments is warranted to confirm and extend these associations, and to better inform targeted prevention and intervention strategies.

## Supplementary Information

Supplementary Material 1.

## Data Availability

The datasets generated and/or analyzed during the current study are not publicly available but are available from the corresponding author on reasonable request.
