# Supplementary material for: Childhood trauma and disordered eating behaviors in youth: examining individual types, cumulative numbers, and latent patterns
Source: Child Adolesc Psychiatry Ment Health. 2025 Aug 1;19:91. doi: 10.1186/s13034-025-00928-y (PMC12317485; doi:10.1186/s13034-025-00928-y)
Supplement: Supplementary file 1 — Supplementary Material 1. [file 13034_2025_928_MOESM1_ESM.docx]

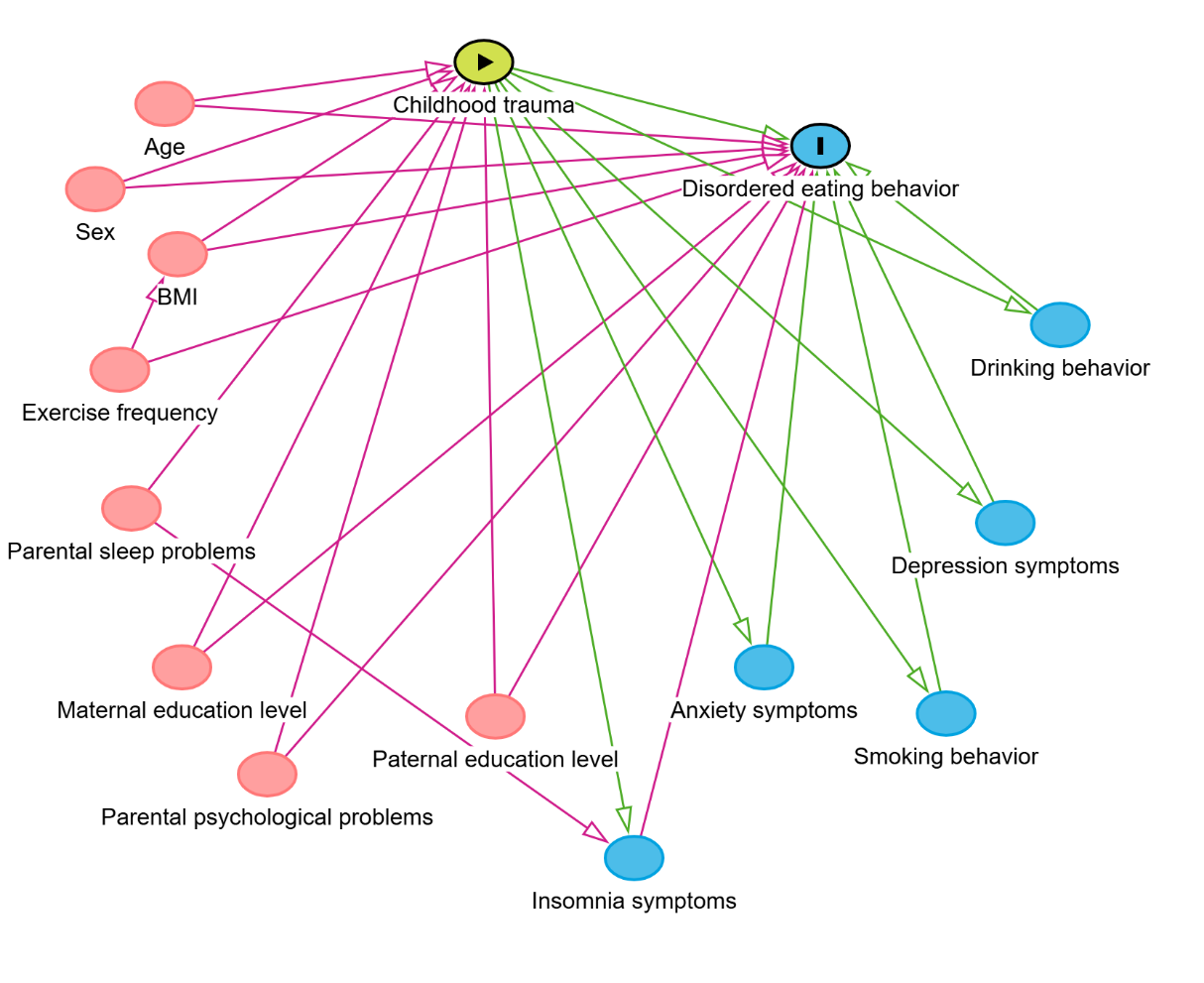


**Figure S1.** Directed Acyclic Graph illustrating hypothesized relationships among childhood trauma, disordered eating behaviors, and covariates.

| Table S1 Model fit indices derived from latent class analysis on models with 1-5 classes | | | | | | | | |
| --- | --- | --- | --- | --- | --- | --- | --- | --- |
| Model | Loglikelihood | AIC | BIC | SSABIC | Entropy | LMR(*p*) | BLRT(*p*) | Class counts and proportions |
| 1 | -6662.605 | 13335.210 | 13365.903 | 13350.016 | - | - | - |  |
| 2 | -6042.925 | 12107.850 | 12175.375 | 12140.423 | 0.713 | <0.0001 | <0.0001 | 969/2455 (0.28/0.72) |
| **3** | **-5893.708** | **11821.417** | **11925.773** | **11871.756** | **0.858** | **<0.0001** | **<0.0001** | **149/2179/1096(0.04/0.64/0.32)** |
| 4 | -5883.673 | 11813.345 | 11954.532 | 11881.450 | 0.785 | 0.1280 | <0.0001 | 1448/181/88/1707 (0.42/0.05/0.03/0.50) |
| 5 | -5881.358 | 11820.716 | 11998.734 | 11906.588 | 0.728 | 0.1974 | 0.6667 | 125/56/1070/2092/81(0.04/0.02/0.31/0.61/0.02) |
| Note: AIC=Akaike Information Criterion; BIC=Bayesian Information Criterion; SSABIC=Sample Size Adjusted Bayesian Information Criterion; LMR=Vuong-Lo-Mendell-Rubin likelihood ratio test; BLRT=bootstrapped likelihood ratio test. | | | | | | | | |

| Table S2 Predicted probabilities of childhood trauma types by class membership | | | |
| --- | --- | --- | --- |
|  | Low trauma | High neglect | High trauma |
| 1. Emotional abuse | 0.012 | 0.055 | 0.777 |
| 2. Physical abuse | 0.012 | 0.013 | 0.608 |
| 3. Sexual abuse | 0.024 | 0.031 | 0.334 |
| 4. Emotional neglect | 0.000 | 0.916 | 0.862 |
| 5. Physical neglect | 0.183 | 0.695 | 0.762 |

| Table S3 Differences in covariates and childhood trauma among LCA-identified childhood trauma types | | | | | | |
| --- | --- | --- | --- | --- | --- | --- |
| Variables | Low trauma  (C1) | High neglect (C2) | High trauma (C3) | *F*/*H*/*χ^2^* | Post hoc tests | *p* |
| **Covariates** | | | | | | |
| Age, M (SD) | 18.15 (2.25) | 18.02 (2.27) | 18.19 (2.44) | 1.317 | C3≈C1≈C2 | 0.269 |
| Sex, *n* (%) |  |  |  | 9.492 |  | 0.009 |
| Male | 873 (40.1) | 496 (45.3) | 55 (36.9) |  | C1≈C3<C2 |  |
| Female | 1306 (59.9) | 600 (54.7) | 94 (63.1) |  | C1≈C3>C2 |  |
| BMI |  |  |  | 34.961 |  | <0.001 |
| Under weight | 572 (26.3) | 261 (23.8) | 36 (24.2) |  | C1<C2≈C3 |  |
| Normal weight | 1228 (56.4) | 658 (60.0) | 82 (55.0) |  | C1>C2≈C3 |  |
| Over weight | 379 (17.4) | 177 (16.1) | 31 (20.8) |  | C1≈C2≈C3 |  |
| Exercise frequency, *n* (%) |  |  |  | 27.752 |  | <0.001 |
| No | 845 (38.8) | 498 (45.4) | 80 (53.7) |  | C1<C2≈C3 |  |
| 1-3 days/week | 906 (41.6) | 381 (34.8) | 40 (26.8) |  | C1>C2≈C3 |  |
| >3 days/week | 428 (19.6) | 217 (19.8) | 29 (19.5) |  | C1≈C2≈C3 |  |
| Smoking behavior |  |  |  | 130.697 |  | <0.001 |
| No | 2068 (95.3) | 1011 (93.1) | 106 (71.6) |  | C1>C2>C3 |  |
| Yes | 101 (4.7) | 75 (6.9) | 42 (28.4) |  | C1<C2<C3 |  |
| Drinking behavior |  |  |  | 79.572 |  | <0.001 |
| No | 1791 (82.3) | 883 (80.6) | 78 (52.3) |  | C1≈C2>C3 |  |
| Yes | 385 (17.7) | 213 (19.4) | 71 (47.7) |  | C1≈C2<C3 |  |
| Paternal education level, *n* (%) |  |  |  | 20.951 |  | <0.001 |
| Middle school and below | 995 (45.7) | 570 (52.0) | 78 (52.3) |  | C1<C2≈C3 |  |
| Senior high school | 588 (27.0) | 281 (25.6) | 25 (16.8) |  | C1≈C2>C3 |  |
| College degree and above | 596 (27.4) | 245 (22.4) | 46 (30.9) |  | C1≈C3>C2 |  |
| Maternal education level, *n* (%) |  |  |  | 10.579 |  | 0.032 |
| Middle school and below | 1193 (54.7) | 656 (59.9) | 84 (56.4) |  | C1≈C3, C1<C2, C2≈C3 |  |
| Senior high school | 539 (24.7) | 255 (23.3) | 31 (20.8) |  | C1≈C2≈C3 |  |
| College degree and above | 447 (20.5) | 185 (16.9) | 34 (22.8) |  | C1>C2, C1≈C3, C2≈C3 |  |
| Parental psychological problems, *n* (%) |  |  |  | 107.411 |  | <0.001 |
| No | 2020 (92.7) | 956 (87.2) | 101 (67.8) |  | C1>C2>C3 |  |
| Yes | 159 (7.3) | 140 (12.8) | 48 (32.2) |  | C1<C2<C3 |  |
| Parental sleep problems, *n* (%) |  |  |  | 17.339 |  | <0.001 |
| No | 1600 (73.46) | 798 (72.8) | 86 (57.7) |  | C1≈C2>C3 |  |
| Yes | 579 (26.6) | 298 (27.2) | 63 (42.3) |  | C1≈C2<C3 |  |
| Depression symptoms, Median (IQR) | 5.00 (7.00) | 7.00 (10.00) | 14.00 (10.00) | 198.902 | C1<C2<C3 | <0.001 |
| Anxiety symptoms, Median (IQR) | 4.00 (7.00) | 5.00 (8.00) | 11.00 (9.00) | 153.703 | C1<C2<C3 | <0.001 |
| Insomnia symptoms, Median (IQR) | 5.00 (7.00) | 7.00 (9.00) | 10.00 (9.00) | 116.273 | C1<C2<C3 | <0.001 |
| **Childhood trauma** | | | | | | |
| Childhood trauma total score, M (SD) | 33.94 (5.69) | 48.87 (5.95) | 67.24 (12.87) | 2725.333 | C1<C2<C3 | <0.001 |
| Emotional abuse, *n* (%) |  |  |  | 1300.043 |  | <0.001 |
| No | 2145 (98.4) | 1006 (91.8) | 30 (20.1) |  | C1>C2>C3 |  |
| Yes | 34 (1.6) | 90 (8.2) | 119 (79.9) |  | C1<C2<C3 |  |
| Physical abuse, *n* (%) |  |  |  | 2209.796 |  | <0.001 |
| No | 2153 (98.8) | 1088 (99.3) | 24 (16.1) |  | C1≈C2>C3 |  |
| Yes | 26 (1.2) | 8 (0.7) | 125 (83.9) |  | C1≈C2<C3 |  |
| Sexual abuse, *n* (%) |  |  |  | 545.688 |  | <0.001 |
| No | 2126 (97.6) | 1061 (96.8) | 85 (57.0) |  | C1≈C2>C3 |  |
| Yes | 53 (2.4) | 35 (3.2) | 64 (43.0) |  | C1≈C2<C3 |  |
| Emotional neglect, *n* (%) |  |  |  | 3348.640 |  | <0.001 |
| No | 2179 (100.0) | 0 (0.0) | 20 (13.4) |  | C1>C3>C2 |  |
| Yes | 0 (0.0) | 1096 (100.0) | 129 (86.6) |  | C1<C3<C2 |  |
| Physical neglect, *n* (%) |  |  |  | 824.528 |  | <0.001 |
| No | 1725 (79.2) | 339 (30.9) | 30 (20.1) |  | C1>C2>C3 |  |
| Yes | 454 (20.8) | 757 (69.1) | 119 (79.9) |  | C1<C2<C3 |  |
| Cumulative childhood trauma, *n* (%) |  |  |  | 4209.117 |  | <0.001 |
| 0 | 1644 (75.4) | 0 (0.0) | 0 (0.0) |  | C1>C2≈C3 |  |
| 1 | 503 (23.1) | 289 (26.4) | 0 (0.0) |  | C1≈C2>C3 |  |
| 2 | 32 (1.5) | 724 (66.1) | 11 (7.4) |  | C1<C3<C2 |  |
| ≥3 | 0 (0.0) | 83 (7.6) | 138 (92.6) |  | C1<C2<C3 |  |
| Note. M=Mean; SD=Standard deviation; *n*=Frequencies; %= Percentages; IQR=Interquartile range; BMI=Body mass index. | | | | | | |

| Table S4 Association of childhood trauma with disordered dieting behaviors: using ‘neither behavior’ as the reference category | | | | | | | | |
| --- | --- | --- | --- | --- | --- | --- | --- | --- |
| Variables | Risky restrictive eating only | |  | Binge/purging only | |  | Both behaviors | |
|  | OR (95%CI) | *p* |  | OR (95%CI) | *p* |  | OR (95%CI) | *p* |
| **Individual childhood trauma types** | | | | | | | | |
| Emotional abuse |  |  |  |  |  |  |  |  |
| No | Reference |  |  |  |  |  |  |  |
| Yes | 1.86 (1.02, 2.39) | **0.044** |  | 1.98 (1.08, 3.64) | **0.028** |  | 3.69 (1.71, 7.98) | **0.001** |
| Physical abuse |  |  |  |  |  |  |  |  |
| No | Reference |  |  |  |  |  |  |  |
| Yes | 0.80 (0.36, 1.77) | 0.576 |  | 1.62 (0.80, 3.28) | 0.184 |  | 2.77 (1.24, 6.20) | **0.013** |
| Sexual abuse |  |  |  |  |  |  |  |  |
| No | Reference |  |  |  |  |  |  |  |
| Yes | 1.51 (0.73, 3.12) | 0.263 |  | 0.89 (0.38, 2.10) | 0.791 |  | 1.40 (0.59, 3.30) | 0.447 |
| Emotional neglect |  |  |  |  |  |  |  |  |
| No | Reference |  |  |  |  |  |  |  |
| Yes | 1.07 (0.64, 1.79) | 0.789 |  | 1.26 (0.76, 2.07) | 0.373 |  | 1.41 (0.65, 3.06) | 0.385 |
| Physical neglect |  |  |  |  |  |  |  |  |
| No | Reference |  |  |  |  |  |  |  |
| Yes | 1.32 (0.80, 2.17) | 0.283 |  | 0.99 (0.60, 1.62) | 0.957 |  | 0.88 (0.41, 1.87) | 0.741 |
| **Cumulative childhood trauma types** | | | | | | | | |
| 0 | Reference |  |  |  |  |  |  |  |
| 1 | 1.47 (0.79, 2.73) | 0.221 |  | 0.87 (0.46, 1.65) | 0.661 |  | 0.79 (0.25, 2.54) | 0.696 |
| 2 | 1.80 (0.97, 3.31) | 0.062 |  | 1.86 (1.08, 3.23) | **0.026** |  | 2.25 (0.88, 5.76) | 0.091 |
| ≥3 | 2.15 (1.07, 4.33) | **0.032** |  | 2.21 (1.11, 4.38) | **0.024** |  | 5.24 (2.07, 13.25) | **<0.001** |
| **LCA-identified childhood trauma types** | | | | | | | | |
| Low trauma | Reference |  |  |  |  |  |  |  |
| High neglect | 1.38 (0.85, 2.24) | 0.198 |  | 1.47 (0.92, 2.35) | 0.108 |  | 1.89 (0.88, 4.09) | 0.104 |
| High trauma | 2.10 (1.04, 4.24) | **0.038** |  | 2.39 (1.18, 4.85) | **0.016** |  | 6.79 (2.95, 15.62) | **<0.001** |
| Note. LCA=latent class analysis; Reference=1; CI=confidence interval; The results of multinomial logistic regressions adjusted for age, sex, BMI, exercise frequency, smoking behavior, drinking behavior, paternal and maternal education level, parental psychological and sleep problems, depression symptoms, anxiety symptoms, and insomnia symptoms. | | | | | | | | |

| Table S5 Association of childhood trauma with disordered dieting behaviors: using ‘both behaviors’ as the reference category | | | | | | | | |  |
| --- | --- | --- | --- | --- | --- | --- | --- | --- | --- |
| Variables | Neither behavior | |  | Risky restrictive eating only | |  | Binge/purging only | |  |
|  | OR (95%CI) | *p* |  | OR (95%CI) | *p* |  | OR (95%CI) | *p* |  |
| **Individual childhood trauma types** | | | | | | | | |  |
| Emotional abuse |  |  |  |  |  |  |  |  |  |
| No | Reference |  |  |  |  |  |  |  |  |
| Yes | 0.27 (0.13, 0.59) | **0.001** |  | 0.50 (0.21, 1.23) | 0.131 |  | 0.54 (0.22, 1.33) | 0.178 |  |
| Physical abuse |  |  |  |  |  |  |  |  |  |
| No | Reference |  |  |  |  |  |  |  |  |
| Yes | 0.36 (0.16, 0.81) | **0.013** |  | 0.29 (0.10, 2.93) | **0.016** |  | 0.58 (0.22, 1.53) | 0.273 |  |
| Sexual abuse |  |  |  |  |  |  |  |  |  |
| No | Reference |  |  |  |  |  |  |  |  |
| Yes | 0.72 (0.30, 1.69) | 0.447 |  | 1.08 (0.40, 2.93) | 0.874 |  | 0.64 (0.22, 1.89) | 0.416 |  |
| Emotional neglect |  |  |  |  |  |  |  |  |  |
| No | Reference |  |  |  |  |  |  |  |  |
| Yes | 0.71 (0.33, 1.54) | 0.385 |  | 0.76 (0.32, 1.83) | 0.541 |  | 0.89 (0.37, 2.14) | 0.796 |  |
| Physical neglect |  |  |  |  |  |  |  |  |  |
| No | Reference |  |  |  |  |  |  |  |  |
| Yes | 1.14 (0.53, 2.42) | 0.741 |  | 1.49 (0.63, 3.54) | 0.361 |  | 1.12 (0.47, 2.65) | 0.796 |  |
| **Cumulative childhood trauma types** | | | | | | | | |  |
| 0 | Reference |  |  |  |  |  |  |  |  |
| 1 | 1.25 (0.39, 4.04) | 0.696 |  | 1.86 (0.51, 6.70) | 0.346 |  | 1.09 (0.30, 4.02) | 0.894 |  |
| 2 | 0.44 (0.17, 1.14) | 0.091 |  | 0.80 (0.27, 2.35) | 0.682 |  | 0.83 (0.29, 2.38) | 0.726 |  |
| ≥3 | 0.19 (0.08, 0.48) | <0.001 |  | 0.41 (0.14, 1.22) | 0.108 |  | 0.42 (0.14, 1.25) | 0.119 |  |
| **LCA-identified childhood trauma types** | | | | | | | | |  |
| Low trauma | Reference |  |  |  |  |  |  |  |  |
| High neglect | 0.53 (0.25, 1.14) | 0.104 |  | 0.73 (0.30, 1.74) | 0.472 |  | 0.78 (0.33, 1.85) | 0.567 |  |
| High trauma | 0.15 (0.06, 0.34) | **<0.001** |  | 0.31 (0.12, 0.83) | **0.020** |  | 0.35 (0.13, 0.96) | **0.041** |  |
| Note. LCA=latent class analysis; Reference=1; CI=confidence interval; The results of multinomial logistic regressions adjusted for age, sex, BMI, exercise frequency, smoking behavior, drinking behavior, paternal and maternal education level, parental psychological and sleep problems, depression symptoms, anxiety symptoms, and insomnia symptoms. | | | | | | | | |  |
